# Receptor–ligand pair typing and prognostic risk model for papillary thyroid carcinoma based on single-cell sequencing

**DOI:** 10.3389/fimmu.2022.902550

**Published:** 2022-07-22

**Authors:** Zhe Xu Cao, Xin Weng, Jiang Sheng Huang, Xia Long

**Affiliations:** ^1^ Department of Thyroid Surgery, The Second Xiangya Hospital, Central South University, Changsha, China; ^2^ Hunan Sixth Engineering Company Construction Hospital, Changsha, China; ^3^ Hospital Office, The Second Xiangya Hospital, Central South University, Changsha, China

**Keywords:** papillary thyroid carcinoma, receptor-ligand pair, single-cell sequencing, tumor microenvironment, PD-1 blockade therapy

## Abstract

The papillary thyroid carcinoma (PTC) microenvironment consists of various cancer and surrounding cells, and the communication between them is mainly performed through ligand–receptor (LR) interactions. Single-cell RNA sequencing (scRNA-seq) has been performed to investigate the role of intercellular communication networks in tumor progression. In addition, scRNA-seq can accurately identify the characteristics of immune cell subsets, which is of great significance for predicting the efficacy of immunotherapy. In this study, the cell–cell communication network was analyzed through LR pairs, and a new PTC molecular phenotype was developed based on LR pairs. Furthermore, a risk model was established to predict patient response to PD-1 blockade immunotherapy. The scRNA-seq dataset was obtained from GSE184362, and the bulk tumor RNA-seq dataset was obtained from The Cancer Genome Atlas. CellPhoneDB was used for cellular communication analysis. LR pair correlations were calculated and used to identify molecular subtypes, and the least absolute shrinkage and selection operator (Lasso) Cox regression was used to develop a risk model based on LR pairs. The IMvigor210 and GSE78220 cohorts were used as external validations for the LR.score to predict responses to PD-L1 blockade therapy. A total of 149 LR pairs with significant expression and prognostic correlation were included, and three PTC molecular subtypes were obtained from those with significant prognostic differences. Then, five LR pairs were selected to construct the risk scoring model, a reliable and independent prognostic factor in the training set, test set, and whole dataset. Furthermore, two external validation sets confirmed the predictive efficacy of the LR.score for response to PD-1 blockade therapy.

## Introduction

The incidence of thyroid cancer has continuously increased and has become the most common carcinoma of the endocrine system, accounting for 70% of deaths from endocrine tumors (1). The vast majority of thyroid cancers are papillary thyroid carcinoma (PTC). Early-stage PTC has a good prognosis after an operation, ^131^I radiation, and thyroid-stimulating hormone suppressive therapy. However, approximately 20% of patients have recurrence and metastasis postoperatively, in whom the tumor is commonly poorly differentiated, and the traditional treatment has limited efficacy, which are the main causes of death (2, 3).

Multiple kinase inhibitors targeting the key oncogenic and vascular endothelial growth factor (VEGF) in thyroid cancer can prolong progression-free survival and are currently used for the treatment of refractory thyroid cancer (4). However, due to the rapid development of drug resistance and the occurrence of adverse reactions, the effectiveness of kinase inhibitors is limited. Therefore, a safer and more effective treatment is still required to achieve long-term tumor control. Immunotherapy is known as the fourth tumor therapy after surgery, radiotherapy, and chemotherapy and uses the specificity and efficiency of the patient’s immune system to kill cancer cells (5). Abundant immune cells are observed in the thyroid cancer microenvironment; thus, immunotherapy is a promising option (6). Previous studies have carried out relevant preclinical experiments, and clinical studies on thyroid cancer immunotherapy have made some progress (7).

The tumor microenvironment consists of tumor cells and surrounding immune cells, fibroblasts, and interstitial cells (8). Cellular heterogeneity due to mutation is manifested at the genomic, transcriptomic, and proteomic levels, which act as markers for further cell subtyping. The presence of multiple types of stromal cells can drive the occurrence, proliferation, and invasion of cancer cells through paracrine signals (9); thereby, studying the subtype of various cells in the tumor microenvironment can provide oncogenic clues different from those of innate cancer cells (10).

Currently, conventional next-generation transcriptome sequencing only aims at mixed multiple cell samples, and the unique characteristics of specific cells are often overlooked (11). The single-cell RNA sequencing (scRNA-seq) technology can identify the expression profile of each cell type and even rare cell subgroups in tissues that have not been previously discovered. In the highly complicated tumor microenvironment, the scRNA-seq technology can explore the more detailed role of intercellular communication networks in tumor progression (12).

Therefore, this study used the scRNA-seq dataset for intercellular communication analysis based on ligand–receptor (LR) pairs in patients with PTC, and a new PTC molecular phenotype and mapping of the immune cell infiltration landscape are developed, thereby establishing a risk model as a reference for the intercellular LR pairs mechanism in PTC prognosis and immunotherapeutic response.

## Methods

### Data source and preprocessing

The scRNA-seq dataset GSE184362 from the Gene Expression Omnibus (GEO) database contains 23 samples from 11 patients with PTC, of which seven “primary tumor” samples were used for subsequent analysis (13). The bulk tumor RNA-seq and clinical data of patients with PTC as of January 2022 were downloaded from The Cancer Genome Atlas (TCGA) database, with the following exclusion criteria: 1) incomplete RNA-seq data, 2) incomplete clinical data, and 3) follow-up time of <30 days. Finally, 483 samples were included after screening, and the Ensembl ids were converted to gene symbols. The gene expression level was measured using the RMA correction of the R package “limma”. Fragments per kilobase per million was converted to trans per kilobase of exon model per million mapped reads for scRNA-seq data consistency. In general, enumeration data were expressed as percentages, and the chi-square test was used for between-group comparisons. Continuous data were expressed as the mean and standard deviation for normally distributed data and as median and interquartile ranges for non-normally distributed data. The t-test was used when comparing two groups with a normal distribution and homogeneity of variance. The corrected t-test was used when the normal distribution did not meet the homogeneity of variance, whereas the Mann–Whitney test was used when it did not meet the normal distribution. One-way ANOVA was used to compare multiple groups that conformed to the normal distribution and homogeneity of variance, whereas the Kruskal–Wallis test was used for non-normally distributed data. Statistical significance was determined using a two-tailed *p*-value of <0.05.

### Single-cell RNA sequencing data analysis

The R (version 3.6.3) was utilized for subsequent analysis. The function CreateSeuratObject in the R package “Seurat” was used to create a Seurat object for each sample and generate a gene count matrix. Cells with gene counts of <500 or mitochondrial genome content of >10% were removed, and then doublets in each sample were deleted using DoubletFinder. Data normalization and batch correction were performed using the R package “scTransform” and “Harmony”, respectively. After the top 1,000 highly variable genes (HVGs) were screened from the normalized matrix, significant principal components were identified using the jackstraw function, and principal component analysis (PCA) was performed. Cell projections were visualized using a two-dimensional map with unified manifold approximation and projection and were clustered using the FindClusters function. The FindAllMarkers were used to screen differentially expressed genes (DEGs) for each cell group. The screening criteria were adjusted *p*-value of <0.05 and an average fold change of >0.1.

### Cell subgroup definition and cell–cell communication analysis

Canonical markers for cell subgroup definition were obtained from a previous study, manually annotated based on their expressions, and acquired six cell subgroups (14), which can be referred to in **Table 1**. For a systematic analysis of cell–cell interaction, cellular communication analysis was performed based on CellPhoneDB (15), a public database involving ligands, receptors, and their interactions, and by annotating the membrane, secreted, and peripheral proteins of each cell subgroup at different time points. Cell–cell communication was predicted by the receptor expression on a cell subgroup and the corresponding ligand on another cell subgroup. The cluster labels of all cells were randomly permuted 1,000 times to determine the mean average receptor and ligand expression levels of interacting clusters, which resulted in a null distribution for each LR pair. *p*-Values can be obtained by determining the proportion of means that were higher than the actual one, which represents the cell type-specific likelihood of the corresponding receptor–ligand complexes.

**Table 1:** Six cell subgroups and corresponding canonical markers.

Cell subgroups	Markers
T/natural killer (NK) cells	CD3D, CD3E, CD3G, CD247
B cells	CD79A, CD79B, IGHM, IGHD
Thyrocytes	TG, EPCAM, KRT18, KRT19
Myeloid cells	LYZ, S100A8, S100A9, CD14
Fibroblasts	COL1A1, COL1A2, COL3A1, ACTA2
Endothelial cells	PECAM1, CD34, CDH5, VWF

### Calculation of ligand–receptor pair correlations and identification of molecular subtypes

As the ligand and their corresponding receptor co-expressions are necessary for intercellular communication, the correlation between LR pairs of gene expression was calculated through Pearson’s correlation coefficient of LR pairs and performed using consistent cluster analysis with a Pearson’s correlation coefficient of >0.3 (adjusted *p* of <0.05) to identify molecular subtypes (16). Briefly, after a consistent matrix was generated through the R package “ConensusClusterPlus” and PAM algorithm, in which “Canberra” was used as the metric distance, 500 bootstraps were performed, with each bootstrap sampling comprising 80% of patients. The number of clusters was set from two to 10, with the optimal number of clusters determined by calculating the consistent matrix and cumulative distribution function (CDF).

### Gene set enrichment analysis and functional annotation

To explore the pathways involved in different molecular subtypes, all candidate gene sets in the Molecular Signature Database (MSigDB) (17) were utilized for Gene Set Enrichment Analysis (GSEA). The R package “ClusterProfiler” was used for Gene Ontology (GO) and Kyoto Encyclopedia of Genes and Genomes (KEGG) analysis of DEGs. The GO analysis includes molecular functions, cellular components, and biological processes.

### Tumor immune infiltration analysis

The assessment matrix of relevant tissues from the Estimation of STromal and Immune cells in MAlignant tumors using the expression data (ESTIMATE) (18) platform was downloaded to calculate the proportion of immune cells. In addition, the R package “e1071” and “parallel” and CIBERSORT platform (19) were used to profile immune cell infiltration by immune-related cell markers of each cell type and set the screening criteria as 1,000 random operations and a *p*-value of <0.05 to quantify the relative abundance of immune cells in PTC.

### Establishment of the risk model

The “sample” function in the R was used to randomly split TCGA dataset into training and test sets in a 1:1 ratio, and the R package “glmnet” was used to perform the least absolute shrinkage and selection operator (Lasso) Cox regression and predict the prognosis for patients based on LR pairs. First, the change trajectory of each independent variable was analyzed. The tuning parameter λ is chosen by cross validation. When lambda is small, the result is essentially the least squares estimates. With the gradual increase of λ, the number of variable coefficients tends to gradually increase by 0. Subsequently, 10-fold CIBERSORT validation was used to develop the model, the CI under each λ was analyzed, and the λ at which the model is optimal was determined. To assess the variability and repeatability of the estimates produced by the Lasso Cox regression model, in the R package “survival”, the cox.ph function was used to develop the Cox proportional hazards model. Patients were divided into high- and low-expression groups based on the median score, a Cox proportional hazards model was fitted with patient death as the endpoint, and a hazard ratio was calculated based on the exponential coefficient of the model. The risk score was calculated using the formula LR.Score = ∑betai × Expi, where i refers to the LR pair expression level and beta is the coefficient of the LR pair in the model. Survival curves were drawn using the Kaplan–Meier method for the prognostic analysis, and the log-rank test was used to determine significant differences. The R package “timeROC” was used to generate receiver operating characteristic (ROC) curves over time and analyze the accuracy of the 1-, 3-, and 5-year prediction models based on the area under the curve (AUC). Nomograms were drawn using the “rms” R package.

### External validation of the LR.score model for predicting responses to PD-1 blockade therapy

A total of 348 patients with urothelial carcinoma in the IMvigor210 cohort (20) and 28 patients with melanoma in the GSE78220 cohort (21) showed varying degrees of responses to anti-PD-1 receptor blockers, including complete response (CR), partial response (PR), stable disease (SD), and progressive disease (PD). These two cohorts were used as external validation of the LR.score for predicting responses to PD-1 blockade therapy. Finally, 116 and 27 patients were included in the IMvigor210 and GSE78220 cohorts, respectively, for the next analysis.

## Results

### The single-cell transcriptome landscape of papillary thyroid carcinoma

**Figure 1:**
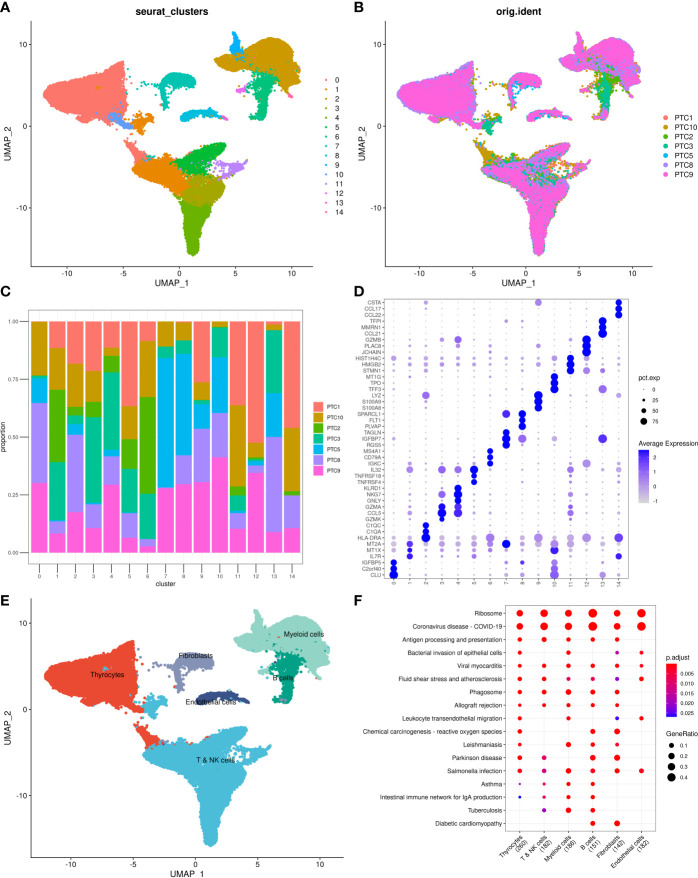
Overview of identified single cells. **(A)** UMAP of cell profiles; different color blocks represent related cell clusters. **(B)** UMAP of cell profiles; different color blocks represent related sample sources. **(C)** Sample distribution of 15 cell clusters. **(D)** Heatmap showing marker genes for each cluster, highlighting selected marker genes for each cluster. **(E)** UMAP of cell profiles; different color blocks represent related cell types. **(F)** Function enrichment of markers for cell subsets. UMAP, Uniform Manifold Approximation and Projection.

Before the quality control, the correlation between the number of unique molecular identifiers (UMIs) and mitochondrial genes and mRNAs was examined. The number of UMIs was not significantly correlated with mitochondrial genes (**Figure S1A**) but positively correlated with mRNAs (**Supplementary Figure S1B**). Furthermore, the number of mRNAs, mRNA reads, and distribution of mitochondrial and nuclear chromosomal genes were determined as shown in **Supplementary Figure 1C**. The number of most genes was distributed between zero and 6,000, and the percentage of mitochondria was <25%. Further, discrete cells, cells with >10% mitochondrial genes, cells with <500 genes, and potential doublets were filtered, and the gene expression profiles of 46,215 high-quality cells were finally obtained. The statistics of the number of cells in each sample are shown in **Table 2**, and the number of filtered mRNAs, mRNA reads, and the number distribution of mitochondrial and nuclear chromosome genes are shown in **Supplementary Figure 1D**. After the quality control, the top 5,000 HVGs were identified for subsequent analysis (**Supplementary Figure 1E**). Cell features were extracted after batch correction based on these HVGs, and 15 cell subgroups were identified using distance matrices (**Figures 1A, B**, **Supplementary Table 1**), where cluster 7 was mainly derived from patient sample PTC5, and cluster 14 was mainly derived from patient PTC1 (**Figure 1C**). The Kruskal–Wallis test was further used to identify differential genes in each cell group (**Supplementary Table 2**); the heatmap of the gene expression is shown in **Figure 1D**, and the heatmap of six subgroups is shown in **Supplementary Figure 2**. Annotation based on classical cell markers resulted in six cell subgroups (**Figure 1E**, **Table 3**). Further, marker genes of each subgroup were identified, and the KEGG enrichment results of these marker genes are shown in **Figure 1F**.

**Table 2:** Cell number statistics before and after sample quality control.

Sample	Raw cell count	Clean cell count	Percent
PTC1	5,917	5,433	91.82
PTC2	5,100	4,716	92.47
PTC3	7,434	6,522	87.73
PTC5	4,475	3,911	87.40
PTC8	10,522	8,775	83.40
PTC9	10,069	8,960	88.99
PTC10	22,390	7,898	35.27

**Table 3:** Cell type annotation for 14 cell clusters.

Cell type	Cluster	Number of cells
Thyrocytes	0	11,933
T and NK cells	1	7,320
Myeloid cells	2	7,089
T and NK cells	3	4,201
T and NK cells	4	3,716
T and NK cells	5	3,051
B cells	6	2,796
Fibroblasts	7	2,373
Endothelial cells	8	1,152
Myeloid cells	9	813
Thyrocytes	10	686
T and NK cells	11	591
Myeloid cells	12	223
Endothelial cells	13	158
Myeloid cells	14	113

### Intercellular communication networks in papillary thyroid carcinoma

**Figure 2:**
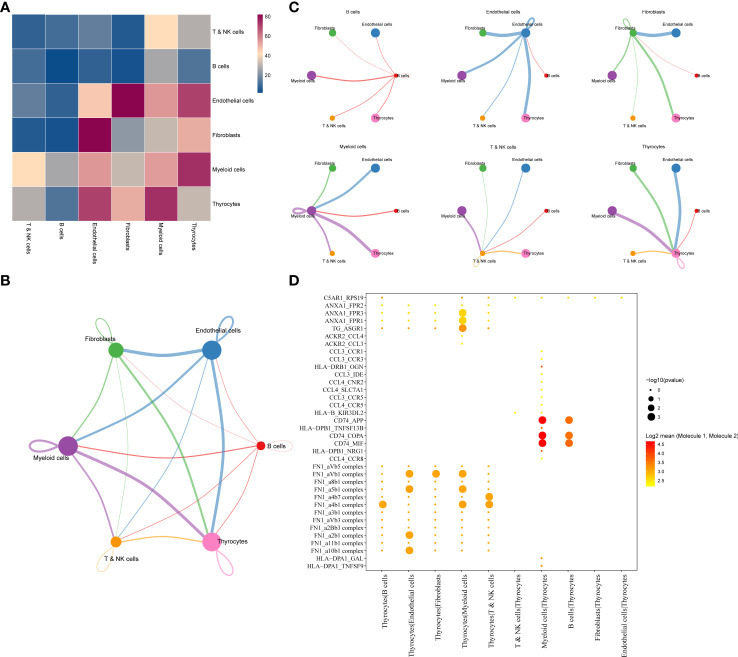
**(A)** LR interactions between different cell subsets. **(B)** Network overview for the interaction between different cell subsets. **(C)** Interactions between each cell subset and other cell subsets. **(D)** Summary of selected significant LR pairs in each cell subset. LR, ligand–receptor.

Results of cell–cell communication analysis show the large-scale interaction of thyrocytes with other cells, among which the strongest interactions were with myeloid and endothelial cells. Furthermore, endothelial cells were found to strongly interact not only with thyrocytes but also with fibroblasts (**Figure 2A**). The interaction network among the six cell subgroups is shown in **Figure 2B**. Among them, thyrocytes, myeloid cells, and endothelial cells had the most cell interactions (**Figure 2C**). Moreover, genes associated with tumor proliferation, metastasis, and progression pathways, including Hedgehog, Notch, TGFβ, WNT, and EGFR, were selected to explore whether significant interaction was observed between cell subgroups. Results suggest that myeloid cells, B cells, and thyrocytes can engage in a range of functional interactions mediated by APP, COPA, and MIF signaling involving the CD74 receptor (**Figure 2D**). LR pairs that significantly differ between cell groups can be referred to in **Supplementary Table 3**.

### Molecular typing based on ligand–receptor pairs

**Figure 3:**
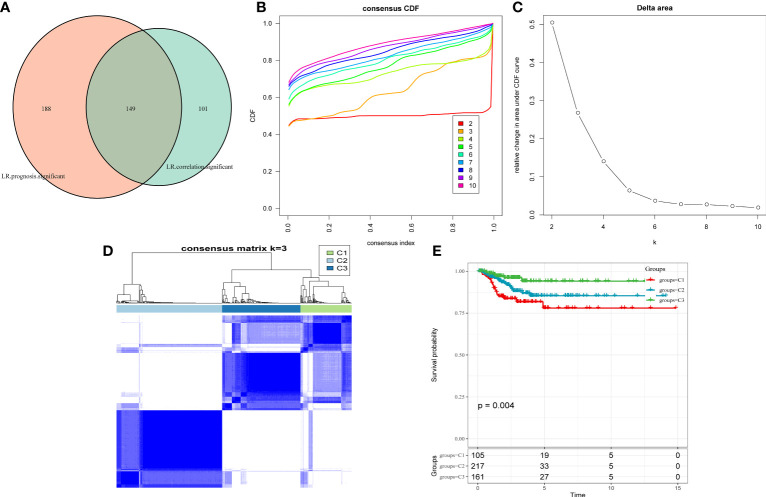
**(A)** Venn diagram of LR pairs with significant expression and prognosis correlation. **(B)** CDF curve of samples from TCGA cohort. **(C)** Delta area curve of samples from TCGA cohort. **(D)** TCGA clustering heatmap of samples from TCGA cohort when consensus k = 3. **(E)** Overall survival curves of molecular subtypes based on LR pairs. LR, ligand–receptor; CDF, cumulative distribution function; TCGA, The Cancer Genome Atlas.

As cell–cell communication greatly differs in various cell subgroups, these differences may lead to activation or inhibition of various pathways, which ultimately result in tumor development and drug resistance. Therefore, LR pairs with significant interactions in different cell subgroups were screened based on Pearson’s correlation coefficient. Here, a total of 250 LR pairs with significant correlations were identified (**Supplementary Table 4**), the total gene expression values of receptors and ligands were used as the expression level of LR pairs, and LR pairs with significant prognostic significance were screened and used for molecular typing. Next, 149 LR pairs with significant expression and prognostic correlation were included (**Figure 3A**), and Cox regression results are shown in **Supplementary Table 5**. According to the CDF, the optimal number of clusters was determined to be three (**Figures 3B**, **C**); hence, three molecular subtypes were finally obtained (**Figure 3D**, **Supplementary Table 6**). Further analysis revealed significant prognostic differences among these three subtypes (**Figure 3E**), with C3 having a better prognosis but C1 having a poorer prognosis.

### Comparison of clinical information in different molecular subtypes

**Figure 4:**
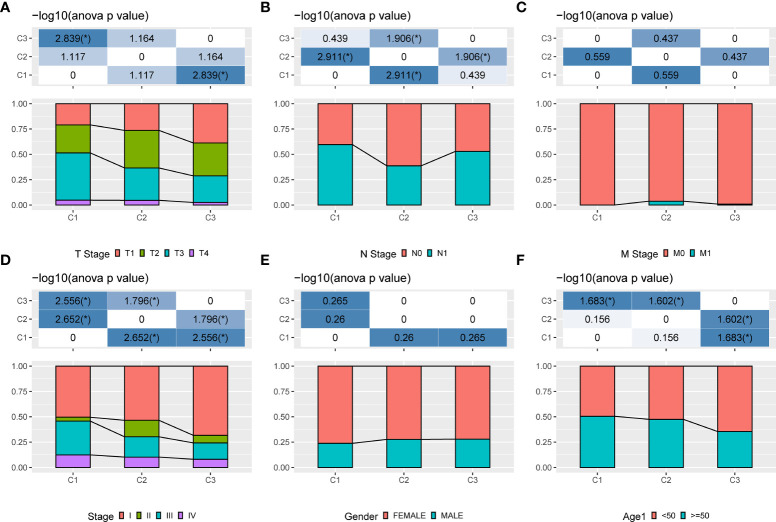
Clinical information of different molecular subtypes. **(A)**: T stage; **(B)**: N stage; **(C)**: M stage; **(D)**: Total stage; **(E)**: Gender; **(F)**: Age.

Three molecular subtypes showed different clinical characteristics in TCGA cohort, and patients with the C1 subtype have a poorer prognosis and a higher TNM stage, with significant differences between C1 and C3 subtypes based on the T stage, overall stage, and age. That is, the proportion of patients with stage III and stage IV in the C1 subtype is higher than that in the C2 and C3 subtypes, and the proportion of patients aged <50 years in the C3 subtype is higher than that in the C1 and C2 subtypes (**Figure 4**).

### Mutational characteristics of different molecular subtypes

**Figure 5:**
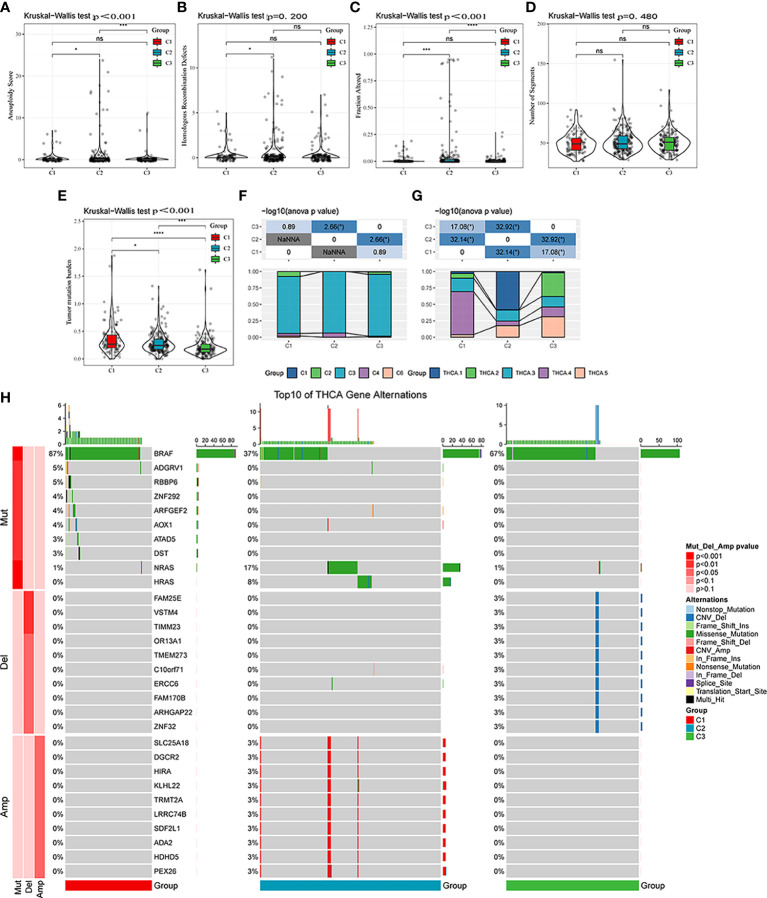
Mutational features of different molecular subtypes. **(A–E)** Comparison of aneuploidy score, homologous recombination defects, altered fraction, number of segments, and tumor mutation burden among different molecular subtypes. **(F)** Comparison of six molecular subtypes and three molecular subtypes. **(G)** Comparison of five molecular subtypes and three molecular subtypes. **(H)** Somatic mutation and copy number variation analysis among the three molecular subtypes. ns, Not statistically significant; *p < 0.05; **p < 0.01; ***p < 0.001; ****p < 0.0001.

Differences in genomic alterations were further explored among these three molecular subtypes. The C1 subtype showed a higher tumor mutation burden (**Figures 5A–E**). Furthermore, information on immune molecular subtypes of TCGA-THCA was obtained from a previous pan-cancer study (14) in which thyroid cancer was classified into six molecular subtypes based on 160 distinct immune signatures; the distribution of these six subtypes per patient can be seen in **Supplementary Figure S3**. Previous subtypes were compared with our defined molecular subtypes, showing that the C3 subtype in the immune molecular subtype accounted for more in our defined C3 subtype, of which Th17 and Th1 characteristics were increasing, and somatic copy number alteration was at lower levels than in other subtypes. Furthermore, the C3 subtype showed the best prognosis, which is consistent with the definition of the molecular C3 subtype in the present study. In addition, the poor prognosis in C1, C2, C4, and C6 immune molecular subtypes was found to be more appropriate in our defined C1 molecular subtype, which is also in line with the poor prognosis in the C1 subtype (**Figure 5F**). Furthermore, five additional molecular subtypes were identified based on consensus clustering in a previous study (22) and were compared with our three molecular subtypes. THCA.4 was higher in the C1 subtype, whereas THCA.2 and THCA.5 were higher in the C3 subtype (**Figure 5G**). Finally, the correlation between gene mutation and copy number variation and molecular subtype was also analyzed, and a significant correlation was found between subtype and gene mutation. Mutation frequencies of BRAF, NRAS, and HRAS are significantly different among subtypes, among which BRAF has a higher mutation frequency in the C1 subtype, whereas NRAS and HRAS have a higher mutation frequency in the C2 subtype. Regarding the copy number variation, the C2 subtype has a higher copy number amplification, whereas the C3 subtype has a relatively high copy number deletion (**Figure 5H**).

### Pathway analysis and immune characterization of different molecular subtypes

**Figure 6:**
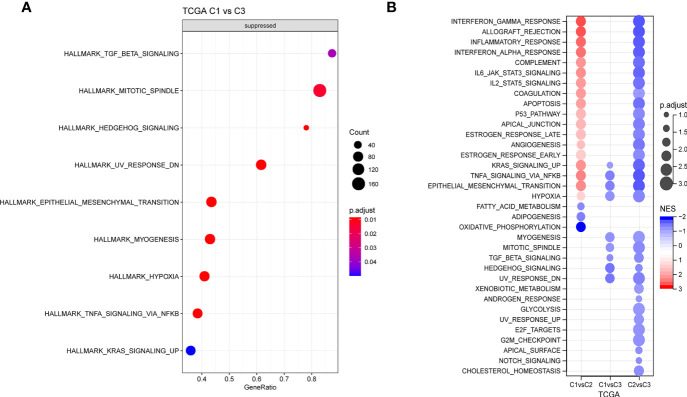
**(A)** GSEA of C1 vs. C3 in TCGA cohort. **(B)** GSEA of different molecular subtypes. GSEA, Gene Set Enrichment Analysis; TCGA, The Cancer Genome Atlas.

**Figure 7:**
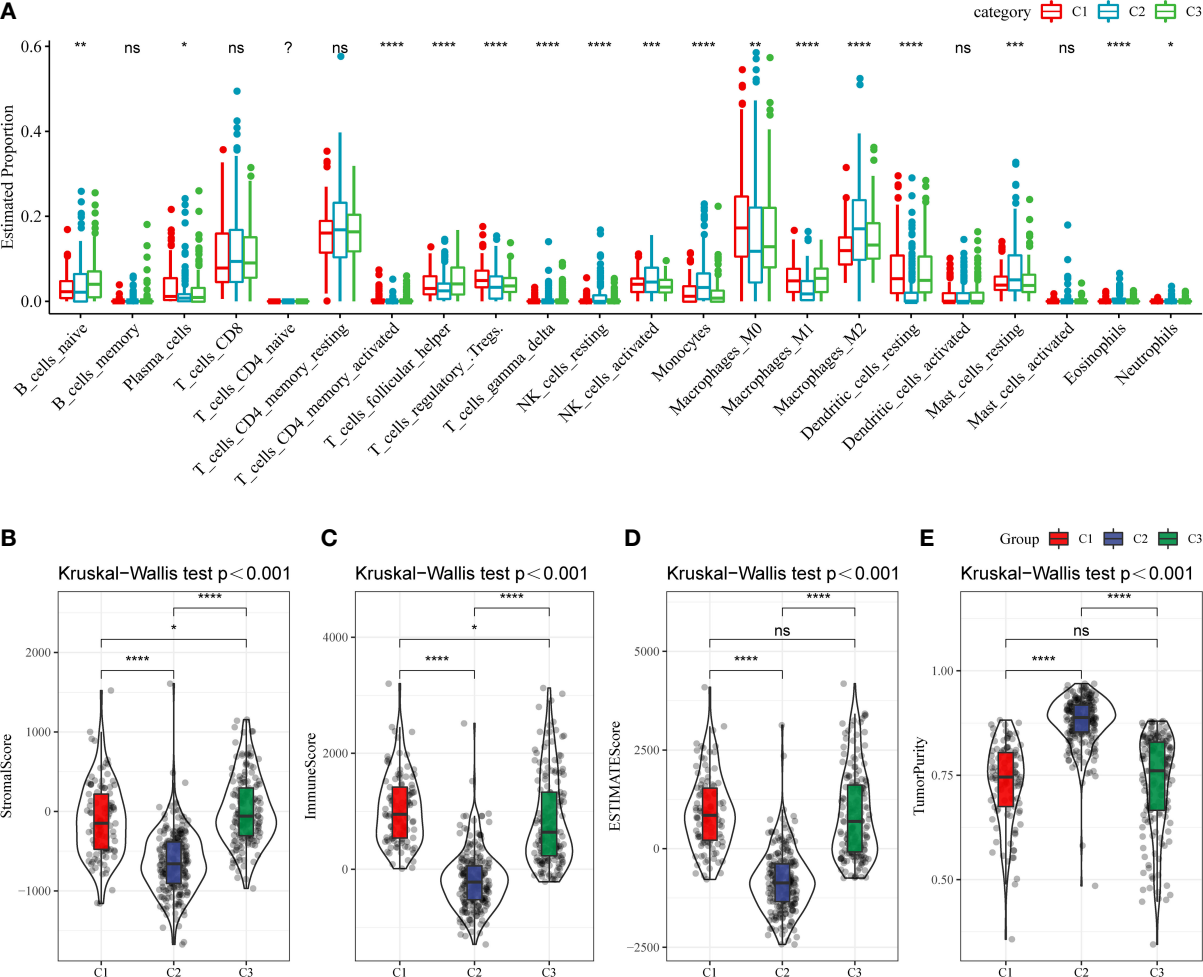
**(A)** The infiltration degree of immune cells in three molecular subtypes. **(B–E)** The proportion of immune cells in three molecular subtypes. ns, Not statistically significant; *p < 0.05; **p < 0.01; ***p < 0.001; and ****p < 0.0001.

Next, whether activated pathways differ among various molecular subtypes was analyzed. Thus, nine pathways were found to be more significantly enriched in the C1 subtype than in the C3 subtype (**Figure 6A**). Furthermore, differences in activated pathways were compared among three molecular subtypes (**Figure 6B**), and results showed that some immune-related pathways were activated in the C1 subtype than in the C2 subtype. Therefore, to further elucidate differences in the immune microenvironment of various subtypes, the infiltration degree of immune cells in the three molecular subtypes was assessed (**Figure 7A**), and significant differences were observed in the degree of most immune cell infiltration among our defined three molecular subtypes. In addition, ESTIMATE was used to evaluate the proportion of immune cells (**Figures 7B**–**E**). “ImmuneScore” was higher in both C1 and C3 subtypes than in the C2 subtype. However, C1 had a poor prognosis, whereas C3 had the opposite.

### Establishment of the risk model based on the ligand–receptor pair score

**Figure 8:**
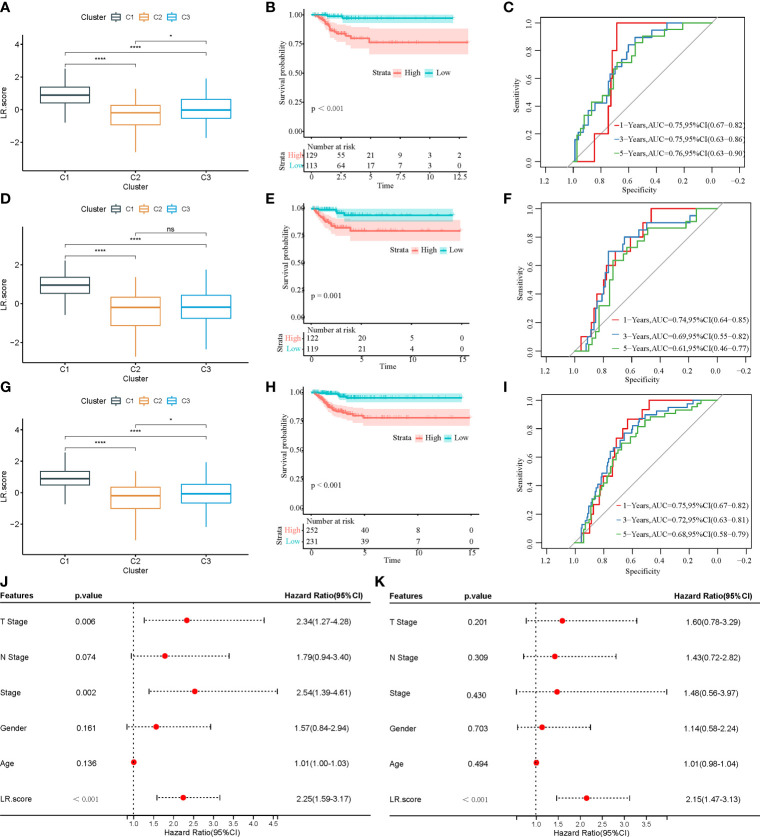
**(A)** LR.score differences in different subtypes in the training set. **(B)** Survival analysis between high and low LR.score groups in the training set. **(C)** The predictive value of LR.score in 1-, 3-, and 5-year overall survival in the training set. **(D)** LR.score differences in different subtypes in the test set. **(E)** Survival analysis between high and low LR.score groups in the test set. **(F)** The predictive value of LR.score in 1-, 3-, and 5-year overall survival in the test set. **(G)** LR.score differences in different subtypes in the entire dataset. **(H)** Survival analysis between high and low LR.score groups in the entire dataset. **(I)** The predictive value of LR.score in 1-, 3-, and 5-year overall survival in the entire dataset. **(J)** Univariate Cox regression model analysis. **(K)** Multivariate Cox regression model analysis.

First, 242 and 241 samples were obtained from the training and test sets, respectively. The clinical and sample information in these two sets is shown in **Table 4**. The chi-square test results showed no significant differences between the two sets. Then, the aforementioned 149 LR pairs with significant expression and prognostic correlation were selected, using Lasso regression to further incorporate them in the training set to reduce the number of genes for developing the risk model (**Supplementary Figure 4A**), and the CI under each λ is shown in **Supplementary Figure 4B**. The model was considered optimal when λ = 0.0218. Thus, five LR pairs were selected when lambda = 0.0218 as the key LR pairs, namely, “ACKR2_CCL14”, “CCL3_CCR1”, “EPHA1_EFNA4”, “HLA-E_KLRC1”, and “LAIR1_LILRB4”; the Cox regression coefficient of these five LR pairs is shown in **Supplementary Figure 4C**. Next, an LR pair score model was created based on these five LR pairs. The LR.score of the C1 molecular subtype is found to be significantly higher than that of the C2 and C3 subtypes (**Figure 8A**). To further evaluate the clinical relevance of the LR pair score, patients were divided into two groups according to low and high LR.scores; i.e., an LR.score of >0 indicates that a patient belongs to the high LR.score group; otherwise, the low LR.score group. The low LR.score group in the training set shows a significantly better prognosis (**Figure 8B**). The AUC values of the time-dependent ROC curve in the LR.score group are 0.75, 0.75, and 0.76 for 1-, 3-, and 5-year overall survival, respectively (**Figure 8C**). Further, the reliability of the LR.score was validated using the test set and the entire dataset (**Figures 8D**, **G**). Patients with low LR.scores in both the test set and the entire dataset were found to have a significant survival benefit (**Figures 8E**, **H**). The AUC of the time-dependent ROC curve is also satisfactory (**Figures 8F**, **I**). To verify whether the LR.score can be used as an independent prognostic indicator, Cox regression analysis was performed based on clinical features and LR.score in TCGA-THCA dataset and found that the LR.score is a reliable and independent factor to evaluate patient prognosis (**Figures 8J**, **K**).

**Table 4:** Comparison of clinical data in of training set, test set, and the entire dataset. .

Characteristics	TCGA-Train (N = 242)	TCGA-Test (N = 241)	Total (N = 483)	*p*-Value
T stage				0.51
T1	71 (14.76%)	70 (14.55%)	141 (29.31%)	
T2	74 (15.38%)	87 (18.09%)	161 (33.47%)	
T3	87 (18.09%)	73 (15.18%)	160 (33.26%)	
T4	9 (1.87%)	10 (2.08%)	19 (3.95%)	
N stage				0.53
N0	109 (25.17%)	114 (26.33%)	223 (51.50%)	
N1	110 (25.40%)	100 (23.09%)	210 (48.50%)	
M stage				0.88
M0	147 (52.69%)	127 (45.52%)	274 (98.21%)	
M1	2 (0.72%)	3 (1.08%)	5 (1.79%)	
Stage				0.18
I	146 (30.35%)	132 (27.44%)	278 (57.80%)	
II	19 (3.95%)	32 (6.65%)	51 (10.60%)	
III	54 (11.23%)	50 (10.40%)	104 (21.62%)	
IV	21 (4.37%)	27 (5.61%)	48 (9.98%)	
Age				0.34
< 50	141 (29.19%)	129 (26.71%)	270 (55.90%)	
≥ 50	101 (20.91%)	112 (23.19%)	213 (44.10%)	
Gender				0.25
Female	183 (37.89%)	170 (35.20%)	353 (73.08%)	
Male	59 (12.22%)	71 (14.70%)	130 (26.92%)	

### Correlation between the LR.score and clinical and immune-related features

**Figure 9:**
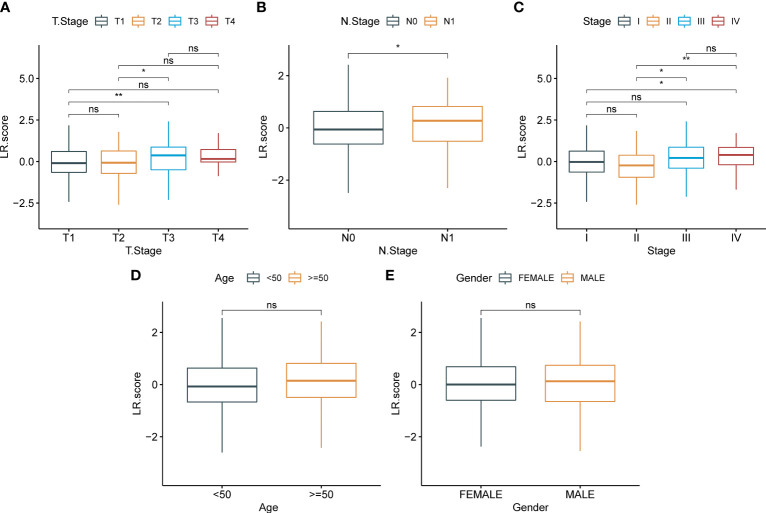
Correlation between LR.score and clinical features. **(A)**: T stage; **(B)**: N stage; **(C)**: Total stage; **(D)**: Age; **(E)**:Gender.

**Figure 10:**
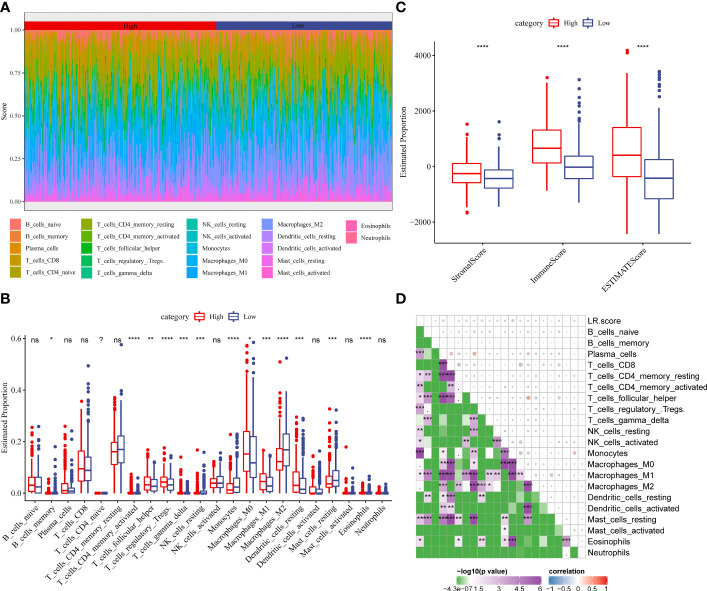
**(A)** Score of 22 immune cell infiltration in different LR.score groups. **(B)** Proportion of 22 immune cell infiltration in different LR.score groups. **(C)** Differences in the distribution of immune cell scores in different LR.score groups. **(D)** Correlation between LR.score and 22 immune cells.

To examine the correlation between the LR.score and clinical characteristics of PTC, the differences in the LR.score were analyzed between various TNM grades and clinical stages in the TCGA-THCA dataset (**Figures 9A**–**E**). Further, we analyzed the infiltration of 22 immune cell types in different LR.score groups (**Figures 10A**, **B**). Overall, the enrichment of most immune cells in the high LR.score group is significantly higher than that in the low LR.score group. Furthermore, differences in the distribution of immune cell scores were compared in various LR.score groups, and the StromalScore, ImmuneScore, and ESTIMATEScore in the high LR.score group were found to be significantly higher than those in the low LR.score group (**Figure 10C**). Moreover, the correlation between LR.score and 22 immune cell types was explored, and the LR.score is positively correlated with T_cells_follicular_helper, T_cells_regulatory_Tregs, and Plasma_cells but negatively correlated with Monocytes and Mast_cells_resting (**Figure 10D**).

### The LR.score model predicts responses to PD-1 blockade immunotherapy

**Figure 11:**
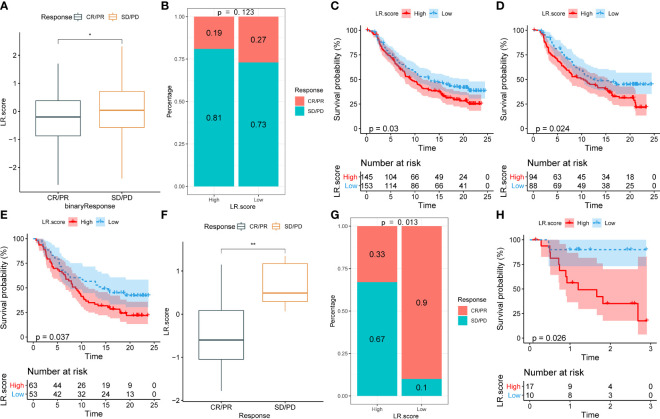
**(A)** Differences in LR.score between immunotherapy response groups in IMvigor210 cohort. **(B)** Differences in immunotherapy response between LR.score groups in IMvigor210 cohort. **(C)** Prognostic difference between LR.score groups in IMvigor210 cohort. **(D)** Prognostic difference between LR.score groups of early-stage patients in IMvigor210 cohort. **(E)** Prognostic difference between LR.score groups of late-stage patients in IMvigor210 cohort. **(F)** Differences in LR.score between immunotherapy response groups in GSE78220 cohort. **(G)** Differences in immunotherapy response between LR.score groups in GSE78220 cohort. **(H)** Prognostic difference between LR.score groups in GSE78220 cohort.

To illuminate the relationship between the LR.score and immunotherapy, we tested its ability to predict responses to anti-PD-1 therapy. We found that in the IMvigor210 cohort, patients with SD/PD have a higher LR.score than those with CR/PR (**Figure 11A**). The comparison between low and high LR.score subgroups also showed that patients in the former have significantly better responses (**Figure 11B**). Patients with low LR.scores acquire significant clinical benefits and prolonged overall survival (**Figure 11C**). The analysis in different stage subgroups showed significant differences in overall survival among patients with different LR.scores in both the stage I+II and stage III+IV subgroups (**Figures 11D**, **E**). Furthermore, the results in the GSE78220 cohort are similar to those in the IMvigor210 cohort (**Figures 11F**–**H**).

## Discussion

Communication between different cells in the tumor microenvironment is primarily performed through LR interactions in soluble or membrane-bound forms (23). Checkpoint inhibitors based on LR interactions have been used as powerful tools for disease treatment (24). In recent years, scRNA-seq has been used to explore intercellular communication within the thyroid cancer microenvironment. Pu et al. found extensive interactions between endothelial cells and a variety of immune cells through scRNA-seq of thyroid cancer tissues, and their intercellular communication was closely related to the activation of the VEGF pathway (13). Pan et al. found a large number of LR interactions between cancer and immune cells, which may affect tumor progression through scRNA-seq of tissues from patients with PTC complicated with Hashimoto’s thyroiditis (25). Peng et al. compared the scRNA-seq landscape of male and female patients with PTC and found significant differences in intercellular communication between different sexes (26). These results suggest that the intercellular communication network-based scRNA-seq can be used to molecularly type PTC and predict prognosis and treatment responses. Hence, we divided PTC patients into three subtypes by LR pairs, and there are significant prognostic differences between patients in different subtypes. Further, we also analyzed the differences in gene mutations between subtypes to explore the molecular mechanism of PTC progression. As the types with a poorer prognosis, the C1 and C2 subtypes have higher mutation frequencies of BRAF and RAS genes. Previous studies have pointed out that the mutations of BRAF and RAS genes can drive PTC to different subtypes, that is, BRAF-like and RAS-like PTC (22). In fact, as the total score of immune cell infiltration in the tumor microenvironment, ImmuneScore is affected by many factors. Although it is positively correlated with the survival of patients with various tumors, there is also some heterogeneity among different tumors. The most noteworthy is the immunogenicity alteration caused by gene mutations. As pointed out by the study by Zhang et al., patients with TP53-mutant breast cancer have a worse prognosis and higher ImmuneScore. This is because TP53-mutant breast cancer has stronger immunogenicity, which also suggests a better response to immunotherapy (27). The C1 subtype has the highest BRAF mutation frequency, and the study by Cen et al. found that BRAF mutant colorectal cancer exhibited more abundant immune cell infiltration and lower tumor purity (28). Similarly, Means et al. also found that BRAF mutation was associated with increased mast cell (MC) density in PTC (29). These results suggest that BRAF-mutant PTC may have a specific tumor microenvironment that alters a patient’s prognosis and response to immunotherapy, which may also affect cell–cell communication that is relevant to our study, and provides us with clues for personalized diagnosis and treatment of PTC according to LR pairs.

Another major advantage of scRNA-seq is that each subset of cells can be analyzed to accurately explore the changes in the tumor microenvironment. Identifying the characteristics of the tumor microenvironment of PTC, especially the characteristics of immune cell subsets and functional status, is of great significance for predicting the immunotherapy efficacy in patients with PTC and proposing feasible adjuvant therapy. T cells, myeloid-derived suppressor cells, tumor-associated macrophages, and other cells play an important role in the dysregulation of the immune microenvironment of thyroid cancer. T cells are divided into CD4+ and CD8+ T cells. CD8+ T cells are mainly differentiated into cytotoxic T lymphocytes, which play a role in the specific killing of target cells. T regulatory cells (Tregs) and T follicular helper cells (Tfh) derived from CD4+ T cells are closely related to immune homeostasis, which not only can maintain peripheral immune tolerance but also can inhibit T cell-mediated immune responses and promote tumor occurrence (30). Moretti et al. reported that Tregs in peripheral blood of patients with PTC were significantly higher than those with thyroid adenoma (31). Gogali et al. reported that Tregs infiltrated more in the PTC tissue than goiter tissue, and the degree of infiltration was positively correlated with disease progression (32). Qian et al. found that the distant metastasis of thyroid cancer was closely related to functionally defective Tfh cells (33). Our results showed that LR.score was positively correlated with the degree of Tregs and Tfh infiltration, which was consistent with the above findings. Similarly, we also found that LR.score was positively correlated with the degree of plasma cell infiltration. However, Kwon et al. pointed out that the infiltration of plasma cells in the thyroid cancer microenvironment indicated a good prognosis (34), which is in contrast to our findings, suggesting that the functional status of immune cells in the tumor microenvironment deserved to be analyzed more precisely. Furthermore, during PTC development, MCs are recruited around cancer tissues and can promote the proliferation, invasion, and metastasis of cancer cells (35). MCs are distributed to differentiated and undifferentiated thyroid cancers, and their density is positively correlated with tumor invasiveness (36). MCs mainly promote cell proliferation through a variety of cytokines such as histamine, CXCL1, GRO-d, and IP-10 (37). The growth-promoting effect of MCs on PTC cells can be inhibited by drugs that promote MC degranulation. Our results showed that LR.score was negatively correlated with MC in the resting phase, suggesting that the inhibition of MCs and their mediators may be a new approach to reverse PTC immune escape.

Immunotherapy has not been the routine treatment of patients with thyroid cancer, and its potential therapeutic effect is unclear. The immune system not only inhibits the occurrence and development of tumors but also promotes tumor growth through the process of immunoediting. The immune escape mechanism of thyroid cancer is complicated and mainly involved the downregulation or loss of function of MHC-1 and upregulation of PD-L1 and BRAF (38). The combination of PD-1 on the T cells and PD-1 on the thyroid cancer cells not only inhibits the proliferation of CD4+ T cells but also induces the apoptosis of CTLs, which plays an important role in the uncontrolled proliferation of cancer cells (39, 40). Therefore, PD-1+ PTC is more likely to progress and relapse (41). The upregulation of BRAF can lead to the high expression of PD-1 on the cancer cells, thereby promoting tumor angiogenesis and malignant proliferation (42, 43).

With the in-depth research on the immune microenvironment of refractory thyroid cancer and its immune escape mechanism, PD-1 blockade immunotherapy is becoming a new treatment for refractory thyroid cancer. Mehnert et al. found that the total efficacy rate of PD-1 inhibitor pembrolizumab in the treatment of patients with advanced PTC and follicular thyroid cancer was 9%, 59% of patients were in stable disease, and 32% of patients were in disease progression (44). Therefore, the current efficacy of PD-1 blockade therapy is very limited, and it is urgent to find good biomarkers and risk models to screen out patients who can benefit from immunotherapy. Therefore, our study verified the ability of the LR.score to predict patients’ responses to PD-1 blockade therapy in IMvigor210 and GSE78220 cohorts. Although additional thyroid cancer cohorts were required for further validation, it is suggested that the risk model based on LR.score has a good predictive ability, providing an important reference for the immunotherapy of thyroid cancer.

## Data availability statement

The datasets presented in this study can be found in online repositories. The names of the repository/repositories and accession number(s) can be found in the article/**supplementary material**.

## Ethics statement

Ethical review and approval was not required for the study on human participants in accordance with the local legislation and institutional requirements. Written informed consent for participation was not required for this study in accordance with the national legislation and the institutional requirements. Written informed consent was not obtained from the individual(s) for the publication of any potentially identifiable images or data included in this article.

## Author contributions

CZX: conceptualization (equal); writing original draft (lead); writing review and editing (equal). WX: formal analysis (supporting); writing review and editing (equal); visualization (lead). HJS: conceptualization (supporting); funding acquisition (lead); writing review and editing (equal); supervision (lead). LX: data curation (lead); funding acquisition (supporting); project administration (lead); writing review and editing (equal). All authors contributed to the article and approved the submitted version.

## Funding

The research is supported by the Key Projects of Intergovernmental International Scientific and Technological Innovation Cooperation (2019YFE0190500) and The Key Guidance Project of Scientific Research Subject of Hunan Provincial Health Commission (202204014372).

## Conflict of interest

The authors declare that the research was conducted in the absence of any commercial or financial relationships that could be construed as a potential conflict of interest.

## Publisher’s note

All claims expressed in this article are solely those of the authors and do not necessarily represent those of their affiliated organizations, or those of the publisher, the editors and the reviewers. Any product that may be evaluated in this article, or claim that may be made by its manufacturer, is not guaranteed or endorsed by the publisher.

## References

[1] Lim H Devesa SS Sosa JA Check D Kitahara CM. Trends in thyroid cancer incidence and mortality in the united states, 1974-2013. JAMA (2017) 317(13):1338–48. doi: 10.1001/jama.2017.2719 PMC8216772 28362912

[2] Sapuppo G Tavarelli M Belfiore A Vigneri R Pellegriti G. Time to separate persistent from recurrent differentiated thyroid cancer: Different conditions with different outcomes. J Clin Endocrinol Metab (2019) 104(2):258–65. doi: 10.1210/jc.2018-01383 30165559

[3] Ibrahim EY Busaidy NL. Treatment and surveillance of advanced, metastatic iodine-resistant differentiated thyroid cancer. Curr Opin Oncol (2017) 29(2):151–8. doi: 10.1097/cco.0000000000000349 28141684

[4] Schlumberger M Tahara M Wirth LJ Robinson B Brose MS Elisei R. Lenvatinib versus placebo in radioiodine-refractory thyroid cancer. N Engl J Med (2015) 372(7):621–30. doi: 10.1056/NEJMoa1406470 25671254

[5] Khalil DN Smith EL Brentjens RJ Wolchok JD. The future of cancer treatment: Immunomodulation, cars and combination immunotherapy. Nat Rev Clin Oncol (2016) 13(5):273–90. doi: 10.1038/nrclinonc.2016.25 PMC5551685 26977780

[6] Papalexi E Satija R. Single-cell rna sequencing to explore immune cell heterogeneity. Nat Rev Immunol (2018) 18(1):35–45. doi: 10.1038/nri.2017.76 28787399

[7] French JD. Immunotherapy for advanced thyroid cancers - rationale, current advances and future strategies. Nat Rev Endocrinol (2020) 16(11):629–41. doi: 10.1038/s41574-020-0398-9 32839578

[8] Ribeiro Franco PI Rodrigues AP de Menezes LB Pacheco Miguel M. Tumor microenvironment components: Allies of cancer progression. Pathol Res Pract (2020) 216(1):152729. doi: 10.1016/j.prp.2019.152729 31735322

[9] Arneth B. Tumor microenvironment. Medicina (Kaunas) (2019) 56(1):15. doi: 10.3390/medicina56010015 PMC7023392 31906017

[10] Galon J Costes A Sanchez-Cabo F Kirilovsky A Mlecnik B Lagorce-Pagès C. Type, density, and location of immune cells within human colorectal tumors predict clinical outcome. Science (2006) 313(5795):1960–4. doi: 10.1126/science.1129139 17008531

[11] Van Loo P Voet T. Single cell analysis of cancer genomes. Curr Opin Genet Dev (2014) 24:82–91. doi: 10.1016/j.gde.2013.12.004 24531336

[12] Shi RL Qu N Luo TX Xiang J Liao T Sun GH. Programmed death-ligand 1 expression in papillary thyroid cancer and its correlation with clinicopathologic factors and recurrence. Thyroid (2017) 27(4):537–45. doi: 10.1089/thy.2016.0228 27825291

[13] Pu W Shi X Yu P Zhang M Liu Z Tan L. Single-cell transcriptomic analysis of the tumor ecosystems underlying initiation and progression of papillary thyroid carcinoma. Nat Commun (2021) 12(1):6058. doi: 10.1038/s41467-021-26343-3 34663816 PMC8523550

[14] Thorsson V Gibbs DL Brown SD Wolf D Bortone DS Ou Yang T-H. The immune landscape of cancer. Immunity (2018) 48(4):812–30.e14. doi: 10.1016/j.immuni.2018.03.023 29628290 PMC5982584

[15] Efremova M Vento-Tormo M Teichmann SA Vento-Tormo R. Cellphonedb: Inferring cell-cell communication from combined expression of multi-subunit ligand-receptor complexes. Nat Protoc (2020) 15(4):1484–506. doi: 10.1038/s41596-020-0292-x 32103204

[16] Wilkerson MD Hayes DN. Consensusclusterplus: A class discovery tool with confidence assessments and item tracking. Bioinformatics (2010) 26(12):1572–3. doi: 10.1093/bioinformatics/btq170 PMC2881355 20427518

[17] Liberzon A Birger C Thorvaldsdóttir H Ghandi M Mesirov JP Tamayo P. The molecular signatures database (Msigdb) hallmark gene set collection. Cell Syst (2015) 1(6):417–25. doi: 10.1016/j.cels.2015.12.004 PMC4707969 26771021

[18] Yoshihara K Shahmoradgoli M Martínez E Vegesna R Kim H Torres-Garcia W. Inferring tumour purity and stromal and immune cell admixture from expression data. Nat Commun (2013) 4:2612. doi: 10.1038/ncomms3612 24113773 PMC3826632

[19] Chen B Khodadoust MS Liu CL Newman AM Alizadeh AA. Profiling tumor infiltrating immune cells with cibersort. Methods Mol Biol (2018) 1711:243–59. doi: 10.1007/978-1-4939-7493-1_12 PMC5895181 29344893

[20] Balar AV Galsky MD Rosenberg JE Powles T Petrylak DP Bellmunt J. Atezolizumab as first-line treatment in cisplatin-ineligible patients with locally advanced and metastatic urothelial carcinoma: A single-arm, multicentre, phase 2 trial. Lancet (2017) 389(10064):67–76. doi: 10.1016/s0140-6736(16)32455-2 27939400 PMC5568632

[21] Hugo W Zaretsky JM Sun L Song C Moreno BH Hu-Lieskovan S. Genomic and transcriptomic features of response to anti-Pd-1 therapy in metastatic melanoma. Cell (2016) 165(1):35–44. doi: 10.1016/j.cell.2016.02.065 26997480 PMC4808437

[22] Cancer Genome Atlas Research N. Integrated genomic characterization of papillary thyroid carcinoma. Cell (2014) 159(3):676–90. doi: 10.1016/j.cell.2014.09.050 PMC4243044 25417114

[23] Ramilowski JA Goldberg T Harshbarger J Kloppmann E Lizio M Satagopam VP. A draft network of ligand-Receptor-Mediated multicellular signalling in human. Nat Commun (2015) 6:7866. doi: 10.1038/ncomms8866 26198319 PMC4525178

[24] Dempke WCM Fenchel K Uciechowski P Dale SP. Second- and third-generation drugs for immuno-oncology treatment-the more the better? Eur J Cancer (2017) 74:55–72. doi: 10.1016/j.ejca.2017.01.001 28335888

[25] Pan J Ye F Yu C Zhu Q Li J Zhang Y. Papillary thyroid carcinoma landscape and its immunological link with hashimoto thyroiditis at single-cell resolution. Front Cell Dev Biol (2021) 9:758339. doi: 10.3389/fcell.2021.758339 34805166 PMC8602800

[26] Peng M Wei G Zhang Y Li H Lai Y Guo Y. Single-cell transcriptomic landscape reveals the differences in cell differentiation and immune microenvironment of papillary thyroid carcinoma between genders. Cell Biosci (2021) 11(1):39. doi: 10.1186/s13578-021-00549-w 33588924 PMC7885238

[27] Zhang Z Hao R Guo Q Zhang S Wang X. Tp53 mutation infers a poor prognosis and is correlated to immunocytes infiltration in breast cancer. Front Cell Dev Biol (2021) 9:759154. doi: 10.3389/fcell.2021.759154 34917611 PMC8669954

[28] Cen S Liu K Zheng Y Shan J Jing C Gao J. Braf mutation as a potential therapeutic target for checkpoint inhibitors: A comprehensive analysis of immune microenvironment in braf mutated colon cancer. Front Cell Dev Biol (2021) 9:705060. doi: 10.3389/fcell.2021.705060 34381786 PMC8350390

[29] Means C Clayburgh DR Maloney L Sauer D Taylor MH Shindo ML. Tumor immune microenvironment characteristics of papillary thyroid carcinoma are associated with histopathological aggressiveness and braf mutation status. Head Neck (2019) 41(8):2636–46. doi: 10.1002/hed.25740 30896061

[30] Tanaka A Sakaguchi S. Regulatory T cells in cancer immunotherapy. Cell Res (2017) 27(1):109–18. doi: 10.1038/cr.2016.151 PMC5223231 27995907

[31] Moretti S Menicali E Voce P Morelli S Cantarelli S Sponziello M. Indoleamine 2,3-dioxygenase 1 (Ido1) is up-regulated in thyroid carcinoma and drives the development of an immunosuppressant tumor microenvironment. J Clin Endocrinol Metab (2014) 99(5):E832–40. doi: 10.1210/jc.2013-3351 24517146

[32] Gogali F Paterakis G Rassidakis GZ Kaltsas G Liakou CI Gousis P. Phenotypical analysis of lymphocytes with suppressive and regulatory properties (Tregs) and nk cells in the papillary carcinoma of thyroid. J Clin Endocrinol Metab (2012) 97(5):1474–82. doi: 10.1210/jc.2011-1838 22399513

[33] Qian G Wu M Zhao Y Li Q Zhang M Cai C. Thyroid cancer metastasis is associated with an overabundance of defective follicular helper T cells. APMIS (2020) 128(8):487–96. doi: 10.1111/apm.13062 32562574

[34] Na KJ Choi H. Immune landscape of papillary thyroid cancer and immunotherapeutic implications. Endocr Relat Cancer (2018) 25(5):523–31. doi: 10.1530/erc-17-0532 29507047

[35] Melillo RM Guarino V Avilla E Galdiero MR Liotti F Prevete N. Mast cells have a protumorigenic role in human thyroid cancer. Oncogene (2010) 29(47):6203–15. doi: 10.1038/onc.2010.348 20729915

[36] Visciano C Liotti F Prevete N Cali G Franco R Collina F. Mast cells induce epithelial-to-Mesenchymal transition and stem cell features in human thyroid cancer cells through an il-8-Akt-Slug pathway. Oncogene (2015) 34(40):5175–86. doi: 10.1038/onc.2014.441 25619830

[37] Pusztaszeri MP Faquin WC Sadow PM. Tumor-associated inflammatory cells in thyroid carcinomas. Surg Pathol Clin (2014) 7(4):501–14. doi: 10.1016/j.path.2014.08.006 26837551

[38] Al-Abdallah A Jahanbani I Mehdawi H Ali RH Al-Brahim N Mojiminiyi O. Down-regulation of the human major histocompatibility complex class I chain-related gene a (Mica) and its receptor is mediated by microrna-146b-5p and is a potential mechanism of immunoediting in papillary thyroid carcinoma. Exp Mol Pathol (2020) 113:104379. doi: 10.1016/j.yexmp.2020.104379 31935378

[39] Cunha LL Marcello MA Vassallo J Ward LS. Differentiated thyroid carcinomas and their B7h1 shield. Future Oncol (2013) 9(10):1417–9. doi: 10.2217/fon.13.89 23651132

[40] Hua D Sun J Mao Y Chen LJ Wu YY Zhang XG. B7-H1 expression is associated with expansion of regulatory T cells in colorectal carcinoma. World J Gastroenterol (2012) 18(9):971–8. doi: 10.3748/wjg.v18.i9.971 PMC3297058 22408358

[41] Ahn S Kim TH Kim SW Ki CS Jang HW Kim JS. Comprehensive screening for pd-L1 expression in thyroid cancer. Endocr Relat Cancer (2017) 24(2):97–106. doi: 10.1530/erc-16-0421 28093480

[42] Bastos AU Oler G Nozima BH Moysés RA Cerutti JM. Braf V600e and decreased nis and tpo expression are associated with aggressiveness of a subgroup of papillary thyroid microcarcinoma. Eur J Endocrinol (2015) 173(4):525–40. doi: 10.1530/eje-15-0254 26338373

[43] Angell TE Lechner MG Jang JK Correa AJ LoPresti JS Epstein AL. Braf V600e in papillary thyroid carcinoma is associated with increased programmed death ligand 1 expression and suppressive immune cell infiltration. Thyroid (2014) 24(9):1385–93. doi: 10.1089/thy.2014.0134 PMC4148060 24955518

[44] Mehnert JM Varga A Brose MS Aggarwal RR Lin CC Prawira A. Safety and antitumor activity of the anti-Pd-1 antibody pembrolizumab in patients with advanced, pd-L1-Positive papillary or follicular thyroid cancer. BMC Cancer (2019) 19(1):196. doi: 10.1186/s12885-019-5380-3 30832606 PMC6399859

