# Supplementary material for: Receptor–ligand pair typing and prognostic risk model for papillary thyroid carcinoma based on single-cell sequencing
Source: Front Immunol. 2022 Jul 22;13:902550. doi: 10.3389/fimmu.2022.902550 (PMC9354623; doi:10.3389/fimmu.2022.902550)
Supplement: Supplementary file 1 [file DataSheet_1.zip › Supplementary Figures.docx]

Supplementary Figures


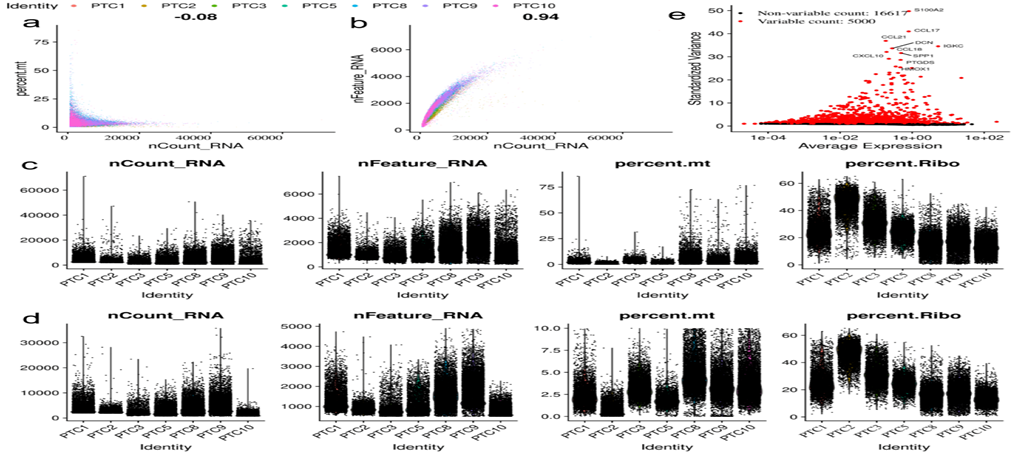


**Supplementary Figure 1 quality control of scRNA-seq data** a: The correlation between the number of UMIs and mitochondrial genes; b: The correlation between the number of UMIs and mRNAs; c: The distribution of mitochondrial and nuclear chromosomal genes; d: The number of filtered mRNAs, mRNA reads and the number distribution of mitochondrial and nuclear chromosome genes; e:The top 5000 HVGs identified for subsequent analysis.


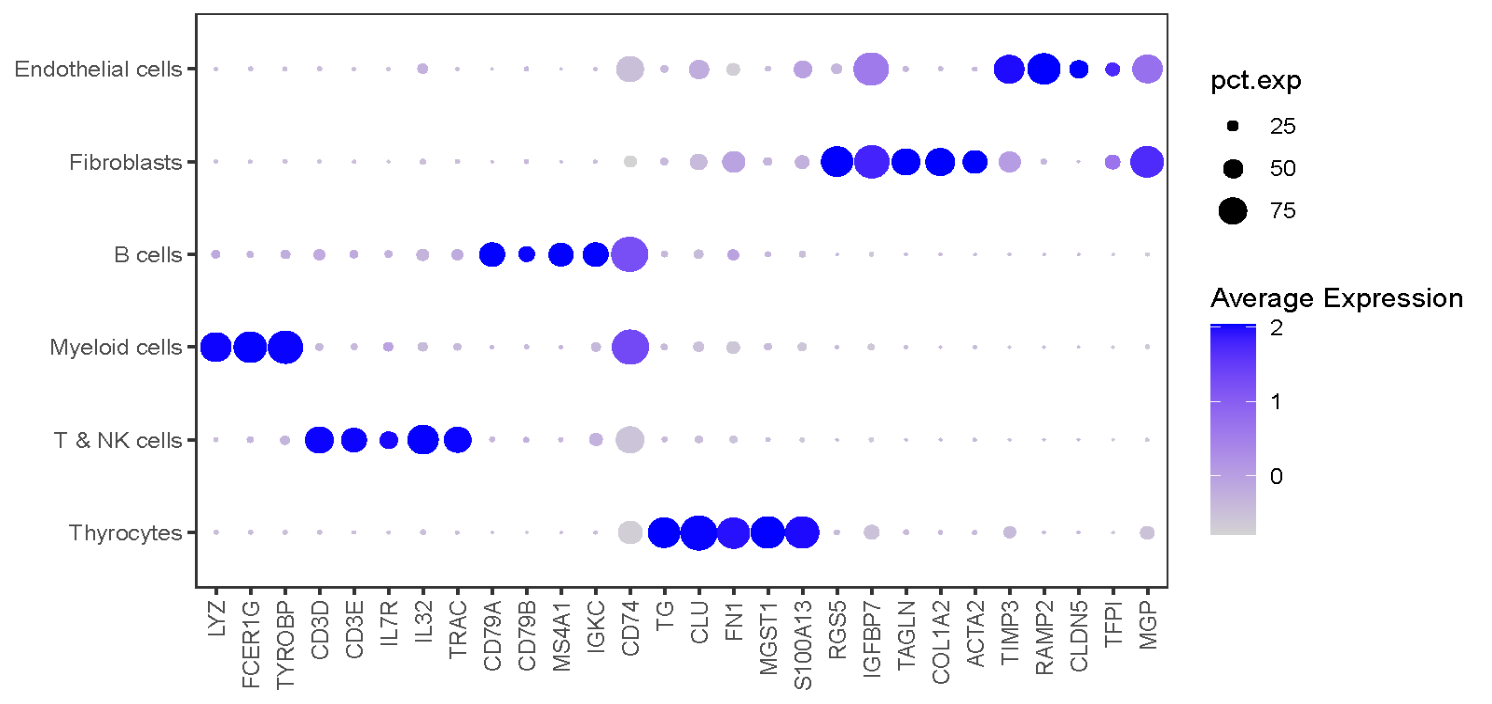


**Supplementary Figure 2**: The heatmap of six subgroups.

**
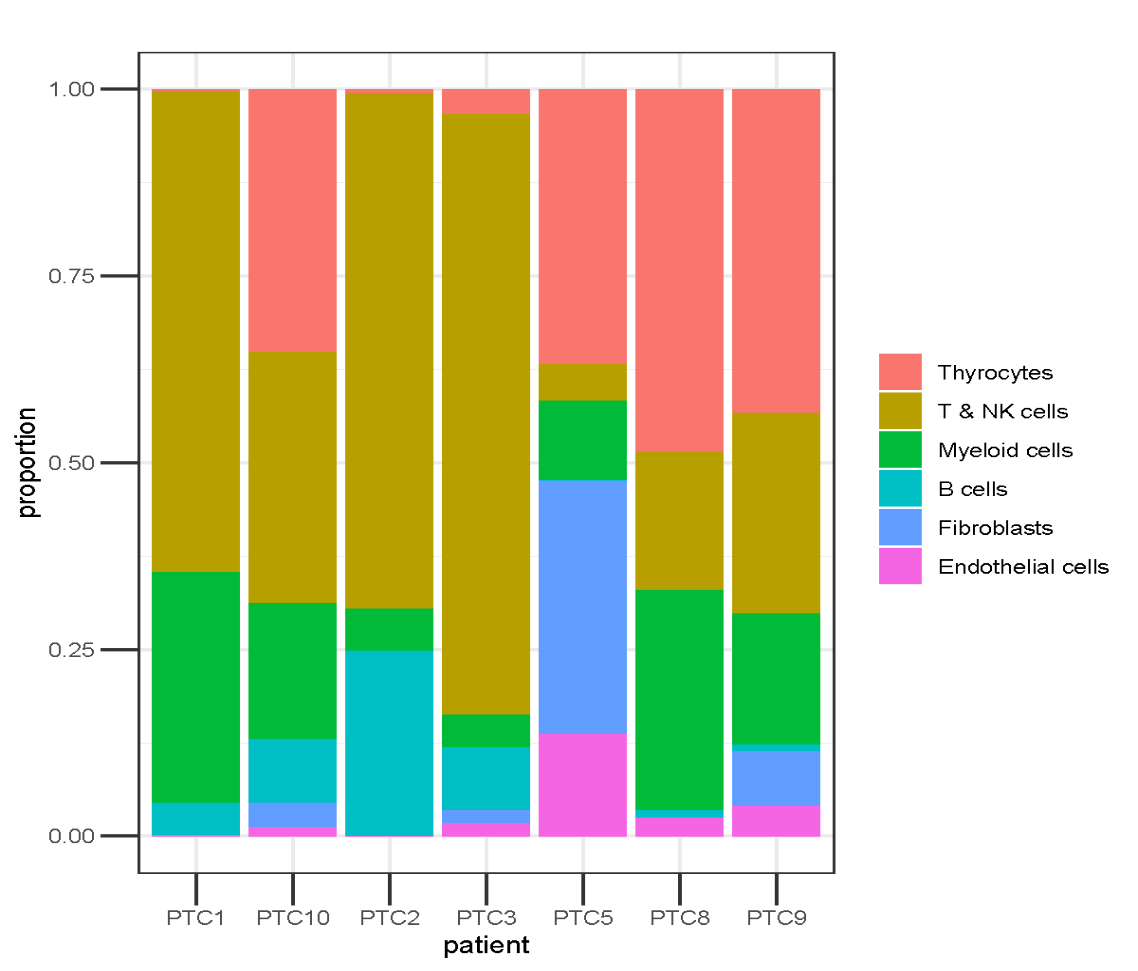
**

**Supplementary Figure 3**: The distribution of six subtypes per patient.

**
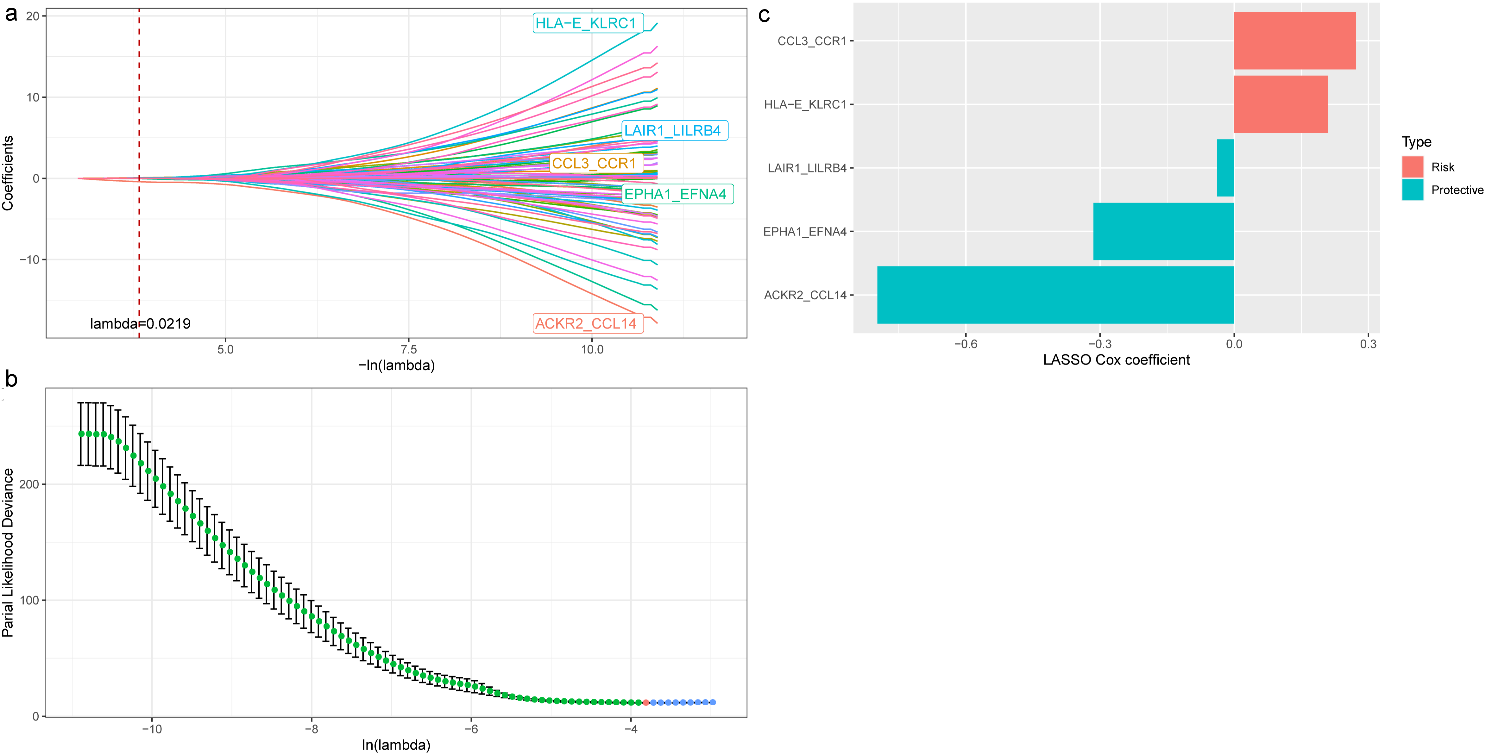
**

**Supplementary Figure 4** a: Lasso regression based on 149 LR-pairs; b: The confidence interval under each λ; c: The Cox regression coefficient of five LR pairs.
